# Biallelic *EZH1* Nonsense Novel Variant in Two Siblings with Neurodevelopmental Disorder and Central Precocious Puberty: A Case Report from a Consanguineous Saudi Family

**DOI:** 10.3390/ijms27167087

**Published:** 2026-08-07

**Authors:** Mohamed Baity, Khalid Alharbi, Eman AlObeid, Albandary AlBakheet, Mohammed Mahnashi, Ali Arishi, Mohamed Tohary, Stefan T. Arold, Dilek Colak, Namik Kaya

**Affiliations:** 1 Translational Genomics Department, Genomic Medicine Center of Excellence, King Faisal Specialist Hospital, and Research Centre (KFSHRC), Riyadh 11211, Saudi Arabia or mbaity@moh.gov.sa (M.B.); esalobeid@kfshrc.edu.sa (E.A.); binbakheet@kfshrc.edu.sa (A.A.); 2 Department of Clincial Laboratory Sciences, College of Applied Medical Sciences, King Saud University, Riyadh 11433, Saudi Arabia; kharbi@ksu.edu.sa; 3 Jazan Specialist Hospital, Jazan 82513, Saudi Arabia; 4 King Fahd Central Hospital, Jazan 82666, Saudi Arabia; mamahnashi@moh.gov.sa (M.M.); aharishi@moh.gov.sa (A.A.); or mtohary@kfshrc.edu.sa (M.T.); 5 Medical Genomics Department, Genomic Medicine Center of Excellence, King Faisal Specialist Hospital, and Research Centre (KFSHRC), Riyadh 11211, Saudi Arabia; 6 Biomedical Sciences Division, KAUST Center of Excellence for Smart Health, King Abdullah University of Science and Technology (KAUST), Thuwal 23955-6900, Saudi Arabia; stefan.arold@kaust.edu.sa; 7 Colak Lab, Research and Innovation, King Faisal Specialist Hospital, and Research Centre, Riyadh 11211, Saudi Arabia

**Keywords:** EZH1, truncation, biallelic variant, nonsense, neurodevelopmental, consanguineous

## Abstract

Neurodevelopmental disorders (NDDs) are a group of conditions that impair the development and function of the central nervous system. Recently, variants in the *EZH1* gene have been associated with neurodevelopmental disorders. Here, using whole-exome sequencing coupled with confirmatory Sanger sequencing, we identified a homozygous nonsense variant in *EZH1* in two affected siblings. Both parents were heterozygous carriers of the variant. The variant is predicted to result in a 44-amino acid C-terminal truncation within the catalytic SET domain, leading to loss of protein function. RT-qPCR analysis revealed significantly reduced *EZH1* mRNA expression in patient-derived peripheral blood cells. The index patient (female) also exhibited elevated gamma-glutamyl transferase (GGT) levels and hypoalbuminemia, whereas the affected male presented with central precocious puberty. This study further expands the clinical, genetic, and molecular spectrum of EZH1-associated neurodevelopmental disorders by demonstrating that reduced *EZH1* expression is consistent with a loss-of-function disease mechanism.

## 1. Introduction

Neurodevelopmental disorders (NDDs) are a group of conditions characterized by impaired development and/or function of the central nervous system. NDDs include intellectual disability (ID), autism spectrum disorder (ASD), developmental delay (DD), and attention-deficit/hyperactivity disorder (ADHD) [1]. The genetic architecture of NDDs is highly heterogenous, involving hundreds of genes and both common and rare variants. In the last decade, the rapid expansion of molecular research and diagnostic technologies has greatly increased the availability of genomic data, leading to improved genetic diagnostic yields and the discovery of hundreds of new genes and variations. Although the pathogenic mechanisms for many of these findings remain incompletely defined, several have been associated with distinctive phenotypic abnormalities in individuals with NDDs [2]. Enhancer of zeste homolog 1 (*EZH1*) is a rare disease-associated gene encoding a component of the noncanonical polycomb repressive complex 2 (PRC2), with roles implicated in multicellular developmental processes, including maintenance of cellular plasticity and embryonic stem cell pluripotency [3]. EZH1 also functions in the mediation of histone H3 lys27 (H3K27) [3]. *EZH1* is broadly expressed across human tissues, with relatively high expression in the brain and gonadal tissues, as demonstrated by expression profiling in the PsychSCREEN database [4]. This expression pattern is consistent with its established roles in maintaining neural cell identity, regulating neurodevelopment, and promoting germ-cell differentiation. In the gonads, EZH1 contributes to epigenetic regulation during oogenesis and spermatogenesis, thereby supporting germ-cell development and reproductive function. Reduced *EZH1* expression may disrupt neural differentiation and neurogenesis and impair germ-cell development, consistently with the neurodevelopmental and endocrine phenotypes associated with EZH1 deficiency [5,6,7]. More recently, pathogenic variants in *EZH1* have been linked to neurodevelopmental disorders [5,8,9]. To our knowledge, two *EZH1* homozygous variants (p.E485X, p.R258X) in six patients from two unrelated consanguineous families were identified in previously undiagnosed NDDs [5]. In this study, we report additional affected individuals from a consanguineous Saudi family harboring a novel homozygous variant, c.2109T>G p.(Tyr703*), in *EZH1*.

## 2. Results

### 2.1. Clinical Features

Patient 1 was an 8-year-old boy who presented with delayed ability to sit and walk, delayed speech and language development, hyperactivity, intellectual disability, motor delay, and precocious puberty (Table 1). Brain magnetic resonance imaging (MRI) was normal, and single-nucleotide polymorphism (SNP) array testing was negative.

**Figure 1:**
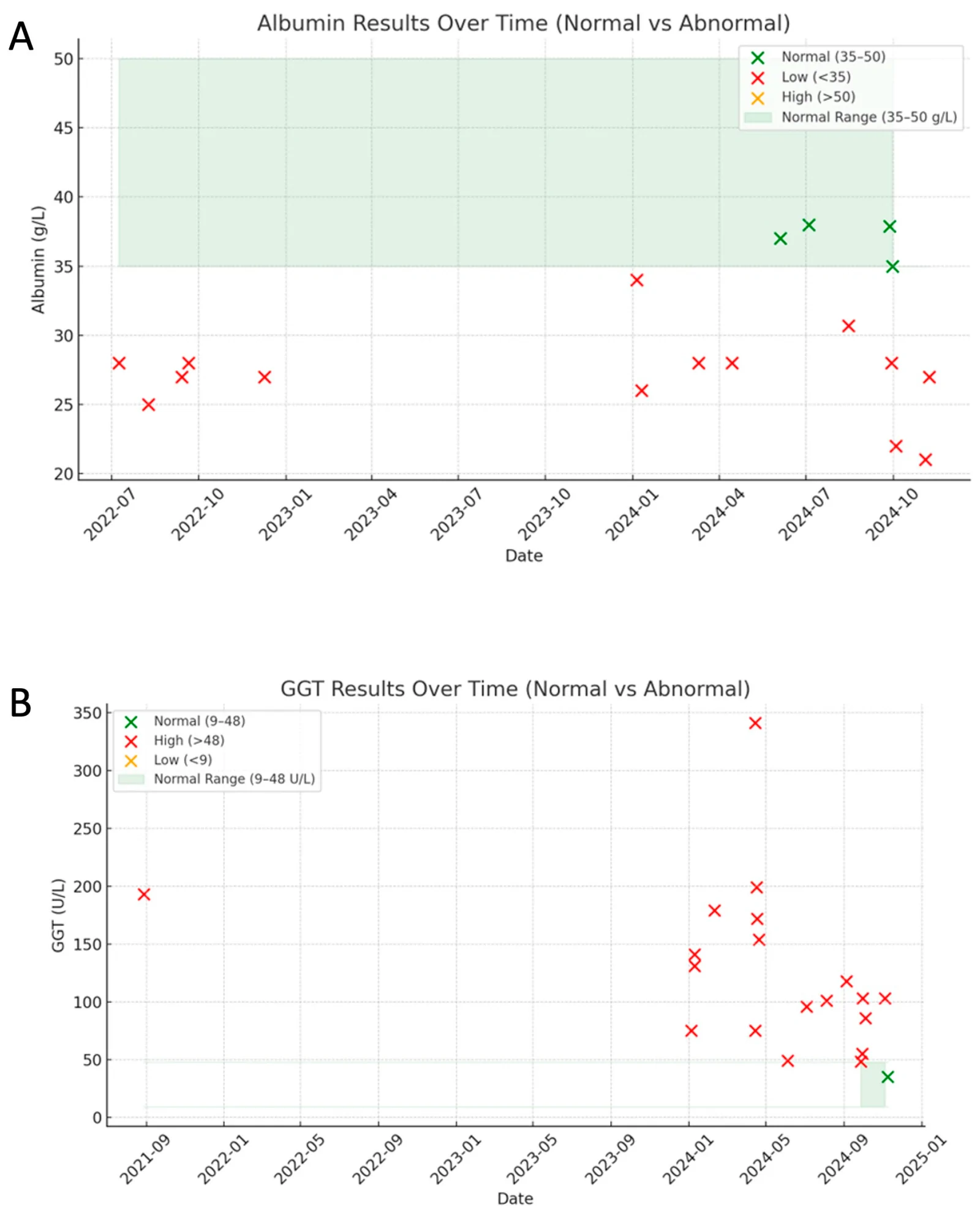
Longitudinal laboratory trends in patient 2. (**A**) Serum albumin measurements over time, with the reference interval shaded (35–50 g/L). (**B**) Serum gamma-glutamyl transferase (GGT) measurements over time, with the reference interval shaded (9–48 U/L). Values are color-coded to indicate results within the reference range (green) versus abnormal (red).

Patient 2 was a 34-year-old woman with complex neurodevelopmental and medical comorbidities. She had a longstanding history of severe mental retardation with significant behavioral impairment, epilepsy, cerebral palsy, and hypertension. Her surgical history included a notable exploratory laparotomy for an acute abdomen due to intestinal adhesions causing small-bowel obstruction. She also had a documented history of developmental ovarian cysts (specified and unspecified), recurrent abdominal pain, constipation, and a retained dental root. She experienced multiple acute events, including bacterial pneumonia, candidiasis, viral infection, cough, and episodes of acute respiratory distress with dyspnea. Over the course of her medical care, laboratory evaluation revealed persistent hypoalbuminemia and elevated gamma-glutamyl transferase (GGT) (Figure 1A,B). Other biochemical panels including renal function, liver enzymes, and hematology were generally within reference limits. She suffered from electrolyte variations and occasional mild anemia. She was negative for hepatitis surface antigen, hepatitis B antibodies, MRSA, C. difficile, and HIV. During her ICU stays and multidisciplinary care, she received therapeutic management including anticoagulants, antihypertensives, broad-spectrum antibodies, systemic antifungals, and anticonvulsants (levetiracetam, phenytoin, diazepam). She also underwent physiotherapy focusing on chest physiotherapy, sitting balance training, and range-of-motion exercises, in addition to nutritional support and home care.

### 2.2. Molecular Analysis Results

**Figure 2:**
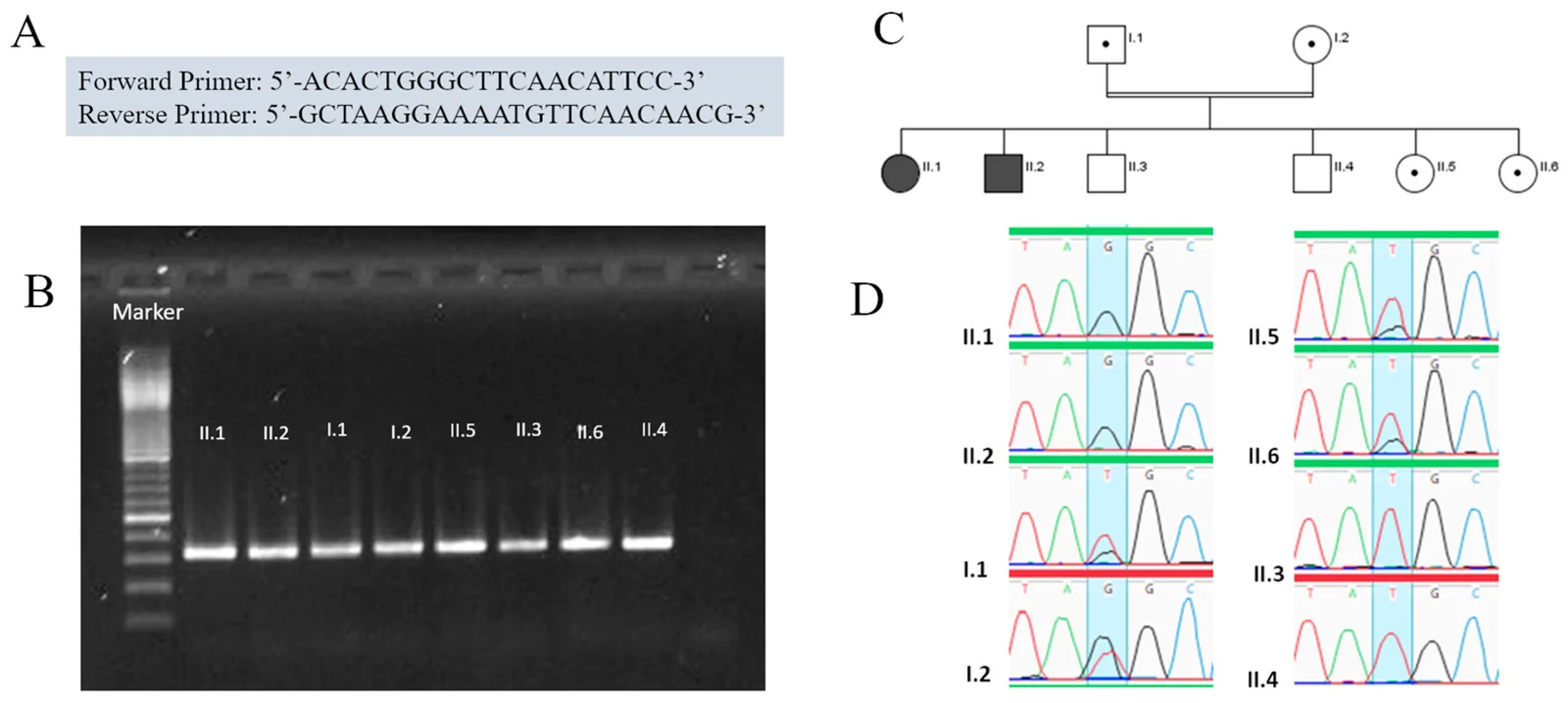
Genetic analysis of the *EZH1* variant: (**A**) Gene-specific primers used in PCR reactions and (**B**) gel electrophoresis showing PCR products of the targeted variant in the gene for all family members. (**C**) The family pedigree of the consanguineous family with two affected children (II.1, II.2) and (**D**) Sanger sequencing results showing segregation of the variant with disease in the family harboring the homozygous nonsense novel variant.

The result of WES analysis for both patients from a consanguineous Saudi family revealed a novel homozygous nonsense variant, in exon 19 of *EZH1* (NM_001321079.1:c.2109T>G:p.(Tyr703*)). The presence of the variant was confirmed on the patients’ DNA, which was amplified by standard PCR using variant-specific primers, followed by Sanger Sequencing (Figure 2A–D). Sanger sequencing of the PCR products from the parents, patients, and remaining family members demonstrated that the segregation of the variant was consistent with the autosomal recessive mode. In brief, the parents were heterozygous and the patients were homozygous; while two sisters were carrier heterozygous, both brothers were wild-type.

The variant was not detected in NCBI ClinVar, GnomeAD, Exome Sequencing Project (ESP), the 1000 Genomes Project, or in-house Saudi exome databases, including CGMdb housed at our Genomic Medicine Center. Based on the American College of Medical Genetics and Genomics (ACMG) guidelines, this variant meets criteria for very strong evidence of pathogenicity (PVS1) consistent with a loss-of-function (LOF) mechanism, and moderate evidence of pathogenicity (PM2) due to absence or extreme rarity in population databases (e.g., ExAC, genomeAD, 1000 Genomes). Collectively, these criteria support classification of the *EZH1* variant as pathogenic. Summary of *EZH1* variants and associated clinical features across different studies are presented in Table 2. 

In silico prediction tools yielded mixed results, with an overall trend toward pathogenicity. CADD (34), BayesDel_addAF (0.53), and BayesDel_noAF (0.52) classified the variant as pathogenic, whereas DANN (0.99) and FATHMM_MKL (0.77) produced results of uncertain significance. Pathogenicity assessment of the *EZH1* variant (c.2109T>G: p.(Tyr703*)) is shown in Table 3. 

**Figure 3:**
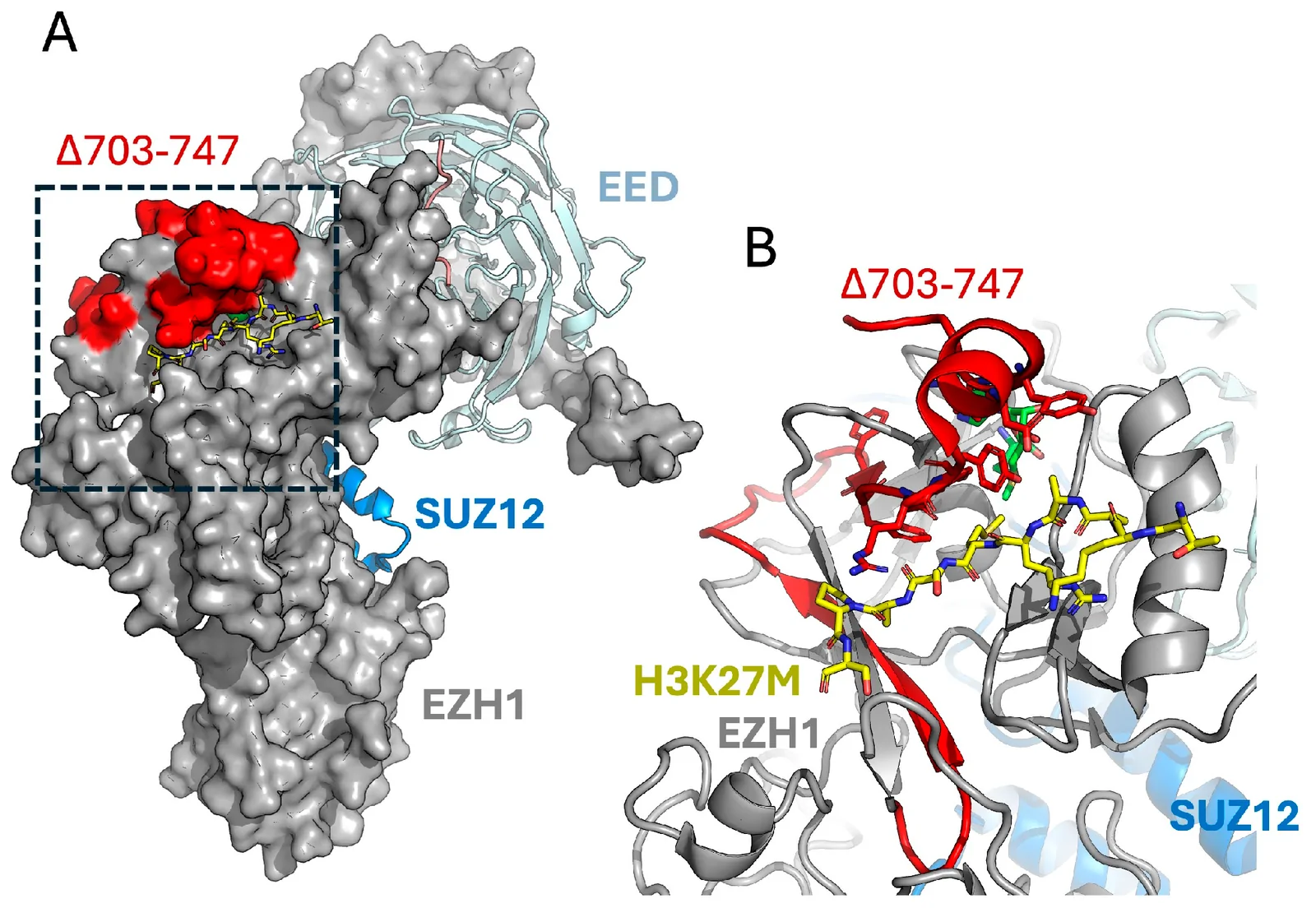
Structural effects of the p.(Tyr703*) variant. (**A**) EZH1 (grey surface) shown in a complex with an H3K27M peptide (stick model, carbons shown in yellow), SUZ12 (blue ribbon) and EED (light blue ribbon). The region deleted in the p.(Tyr703*) variant is shown in red. (**B**) A zoomed-in view of the boxed region in (**A**). Colors are as in (**A**); EZH1 is shown as a ribbon. The SAM is depicted as a stick model, with carbons shown in green. The figure is based on the PDB entry 7TD5.

**Figure 4:**
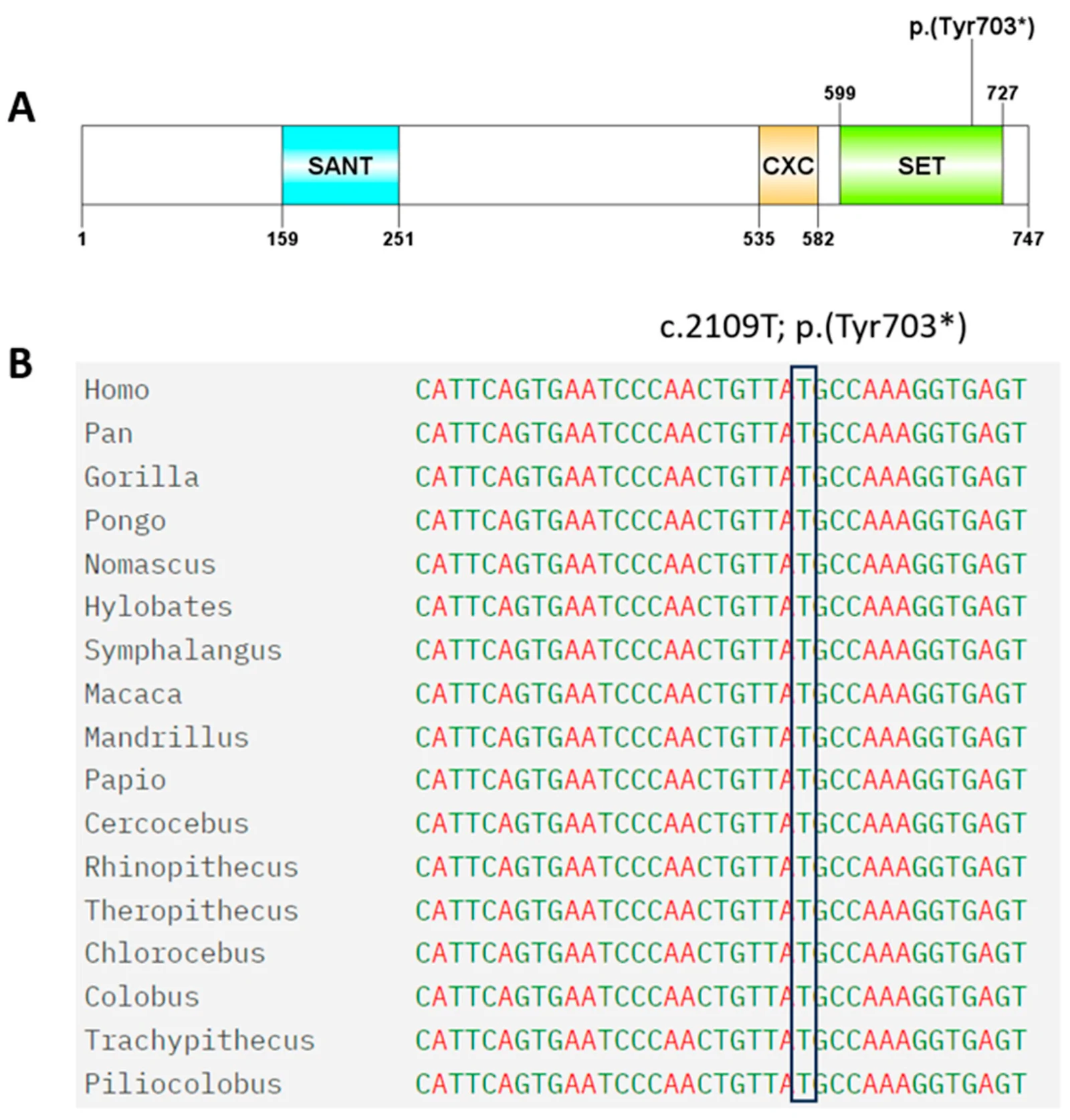
Domains, variant location, and multiple species alignment of amino acid sequences of the EZH1 protein: (**A**) Conserved domains of the EZH1 protein and EZH1 variant p.(Tyr703*) in the SET domain are depicted in the diagram. (**B**) Alignment of amino acid sequences at position 2109 (as indicated by the black rectangle) showing high conservation of the nucleotide in different species.

However, mapping of the variant onto the three-dimensional structure of EZH1 clarified its deleterious effect. The early stop codon leads to a truncation of 44 C-terminal residues. These residues form an integral part of the catalytic SET domain and are essential for its stability and interaction with the cofactor (SAH) and histone substrate (Figure 3A,B). Thus, the variant, located in a highly conserved region and within an important functional domain (Figure 4A,B), results in a complete loss of catalytic function and marked destabilization of the SET domain.

**Figure 5:**
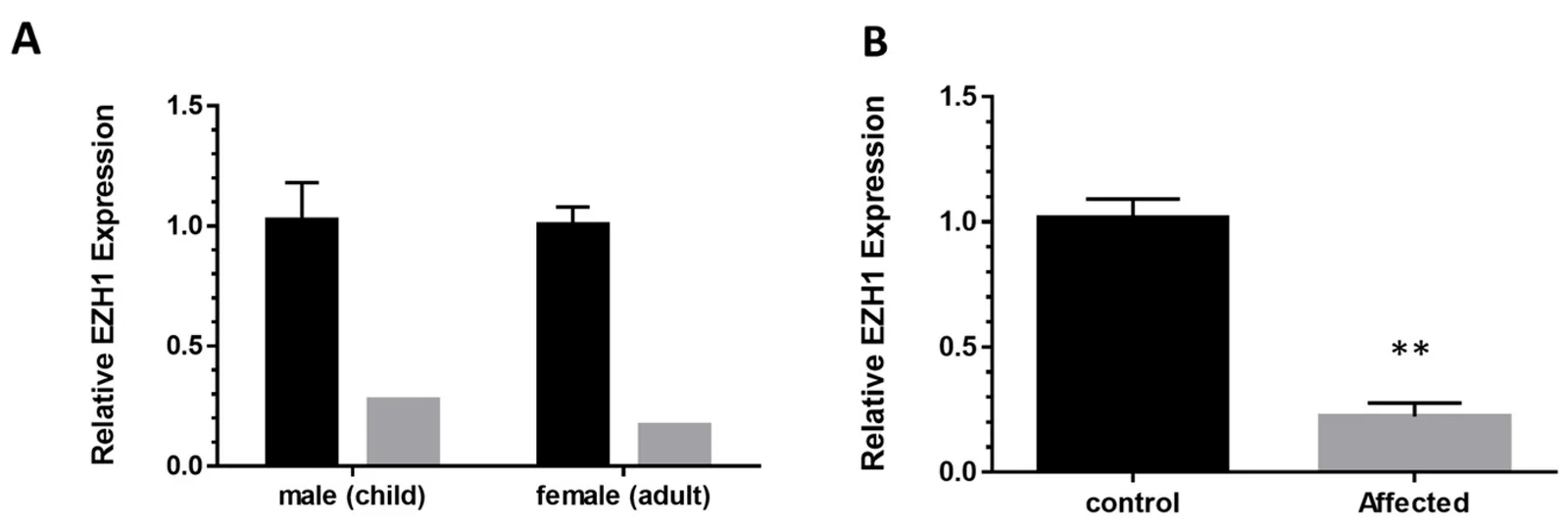
Reduced *EZH1* mRNA expression in affected individuals determined by quantitative RT-PCR. Relative *EZH1* mRNA expression was quantified by RT-qPCR and normalized to that of GAPDH. Relative expression levels were calculated using the 2^−ΔΔCt^ method, with age- and sex-matched control samples as the reference. Data are presented as the mean ± SEM. Black bars represent control samples, whereas grey bars represent affected individuals. (**A**) Relative *EZH1* expression in the affected male child and the affected adult female compared with their respective age- and sex-matched controls. Both affected individuals exhibited markedly reduced *EZH1* expression. (**B**) Combined analysis of control and affected samples demonstrating a significant reduction in *EZH1* mRNA expression in affected individuals compared with controls (two-tailed Student’s *t*-test, *p* = 0.0016). ** *p* < 0.01.

Reverse transcription quantitative PCR (RT-qPCR) was performed on RNA extracted from EBV-transformed lymphoblastoid cells derived from both patients and age- and sex-matched controls. To determine whether expression of *EZH1* is altered in affected individuals, transcript levels were measured using RT–qPCR. Expression values were normalized to that of the housekeeping gene GAPDH, and relative expression levels were calculated using the ΔΔCt method. First, *EZH1* expression was examined in the affected adult female individual and compared with three age- and sex-matched control samples. Following the normalization, *EZH1* mRNA expression was markedly reduced in both affected individuals compared with their respective age- and sex-matched controls (Figure 5A). The reduction was observed consistently in the affected male child and the affected adult female, supporting decreased *EZH1* expression associated with the homozygous nonsense variant. To further evaluate this finding, data from both affected individuals were compared with those from the combined control group. As shown in Figure 5B, EZH1 expression was significantly reduced in affected samples compared with controls (*p* = 0.0016, two-tailed Student’s *t*-test). Overall, these results demonstrate a consistent reduction in EZH1 transcript levels in affected individuals, supporting a loss-of-function mechanism.

## 3. Discussion

*EZH1* is essential in regulating cell differentiation, hematopoiesis, and neurodevelopment [10,11]. Although a direct association between *EZH1* variants and hypoalbuminemia, or elevated GGT has not been reported, indirect mechanisms involving epigenetic regulation by histone methyltransferases and TGF-β-regulated signaling pathways has been implicated in hepatic stellate cell activation and fibrosis [12,13]. In the context of *EZH1* dysregulation, alterations in chromatin-associated regulatory pathways may also influence inflammatory and immune-related processes [14]. Because albumin is a negative acute-phase reactant, chronic inflammation caused by dysregulated *EZH1* may contribute to a reduced serum concentration [15]. GGT is known as an oxidative stress and systemic inflammation marker, and this may provide an indirect link between its elevation and dysregulated *EZH1*. Previous studies have linked *EZH1* to GGT in malignancies [16], in which its elevation may arise from paraneoplastic liver involvement, drug toxicity, and oxidative stress. Decreased albumin and increased GGT levels in patients with NDDs with a mutated *EZH1* variant are most likely caused by secondary conditions such as liver dysfunction, chronic inflammation, or pharmacologic exposure, rather than a primary manifestation of *EZH1* dysfunction.

Recent publications about *EZH1* and its association with neurodevelopmental disorders demonstrated that gain-of-function (GOF) and loss-of-function (LOF) variants cause neurodevelopmental disorders. Jangam et al.’s (2023) [8] study highlighted pathogenic potential in a child with developmental delay and a de novo *EZH1* variant (p.A678G) by showing its GOF activity in *Drosophila melanogaster*. This led to increased H3K27 trimethylation and severe morphological abnormalities. Gracia-Diaz et al. (2023) [5] expanded upon this finding by analyzing 19 individuals with various *EZH1* variants. Ten individuals showed heterozygous missense mutations in which EZH1’s methyltransferase activity increased, while nine individuals showed biallelic truncating mutations (recessive) in which EZH1’s methyltransferase activity was reduced or eliminated (Table 2). 

Among the nine reported individuals, four Saudi patients from a consanguineous family were found to harbor a homozygous nonsense variant (c.1453C>T;p.(Glu485*)). This family shared a common haplotype on chromosome 17 (chr17:40519338–41245021). Moreover, an additional affected child from another consanguineous Saudi family carried the same homozygous variant, suggesting the likely presence of a common genetic founder in the region. *EZH1* variants reported by Gracia-Diaz et al. (2023) [5] and Jangam et al. (2023) [8] have been associated with altered neurogenesis and perturbations in specific cortical neurons, which may disrupt neural network development, ultimately resulting in NDDs.

Regardless of the inheritance pattern or variant type, EZH1-related disorders consistently share common clinical features, including intellectual disability, developmental delay, motor impairment, and language impairment. Jangam et al. [8] reported a patient with a de novo heterozygous missense variant (p.Ala678Gly) who presented with developmental delay, muscle weakness, dyskinetic movements, and hypotonia but showed no evidence of endocrine abnormalities. Gracia-Diaz et al. [5] described 19 affected individuals carrying either biallelic loss-of-function variants or heterozygous gain-of-function variants. Although these individuals exhibited severe and variable neurodevelopmental phenotypes, none were reported to have central precocious puberty (CPP). In contrast, the two affected sisters reported by Okamoto et al. [9] and the two affected siblings in the present study developed CPP in addition to developmental delay, language impairment, intellectual disability, and motor dysfunction. Although CPP appears to be an uncommon manifestation of EZH1-related disorders, it has now been reported in four individuals from two unrelated families. Notably, all four individuals with CPP harbored biallelic loss-of-function variants, whereas no cases of CPP were reported among the 19 individuals described by Gracia-Diaz et al. [5], including those with heterozygous gain-of-function variants. This observation suggests a possible genotype–phenotype correlation, whereby biallelic loss of EZH1 function may predispose affected individuals to hypothalamic–pituitary–gonadal axis dysregulation. Given the established role of EZH1 in epigenetic regulation, disruption of chromatin-mediated gene regulation during brain development and neuronal differentiation may alter the neuroendocrine pathways controlling pubertal onset. Together with the findings of Okamoto et al. [9], our results support the consideration of endocrine evaluation in patients with biallelic loss-of-function *EZH1* variants and expand the clinical spectrum of EZH1-associated neurodevelopmental disorders.

The EZH1 protein consists of three functional domains (SANT, CXC, SET), and the variant c.2109T>G:p.(Tyr703*) in exon 19 sits in a highly conserved region in the SET domain (Figure 4). This domain spans residues 613–728 of EZH1 and provides the methyltransferase activity of the PRC2 complex. The identified novel homozygous *EZH1* (c.2109T>G;p.(Tyr703*)) variant replaces a tyrosine codon with a stop codon at the 703rd amino acid, introducing a premature termination. The resulting truncation is predicted to incapacitate and destabilize the SET domain, abrogating the methyltransferase activity of EZH1. Consistent with an LOF mechanism, RT-qPCR analysis demonstrated a substantial reduction (~4–6 fold) in *EZH1* transcript levels in patient-derived lymphoblastoid cells (Figure 5). This decrease in mRNA abundance likely reflects degradation of the mutant transcript, potentially through nonsense-mediated mRNA decay (NMD), supporting the pathogenic effect of the variant and suggesting that reduced EZH1 dosage contributes to the disease phenotype.

**Figure 6:**
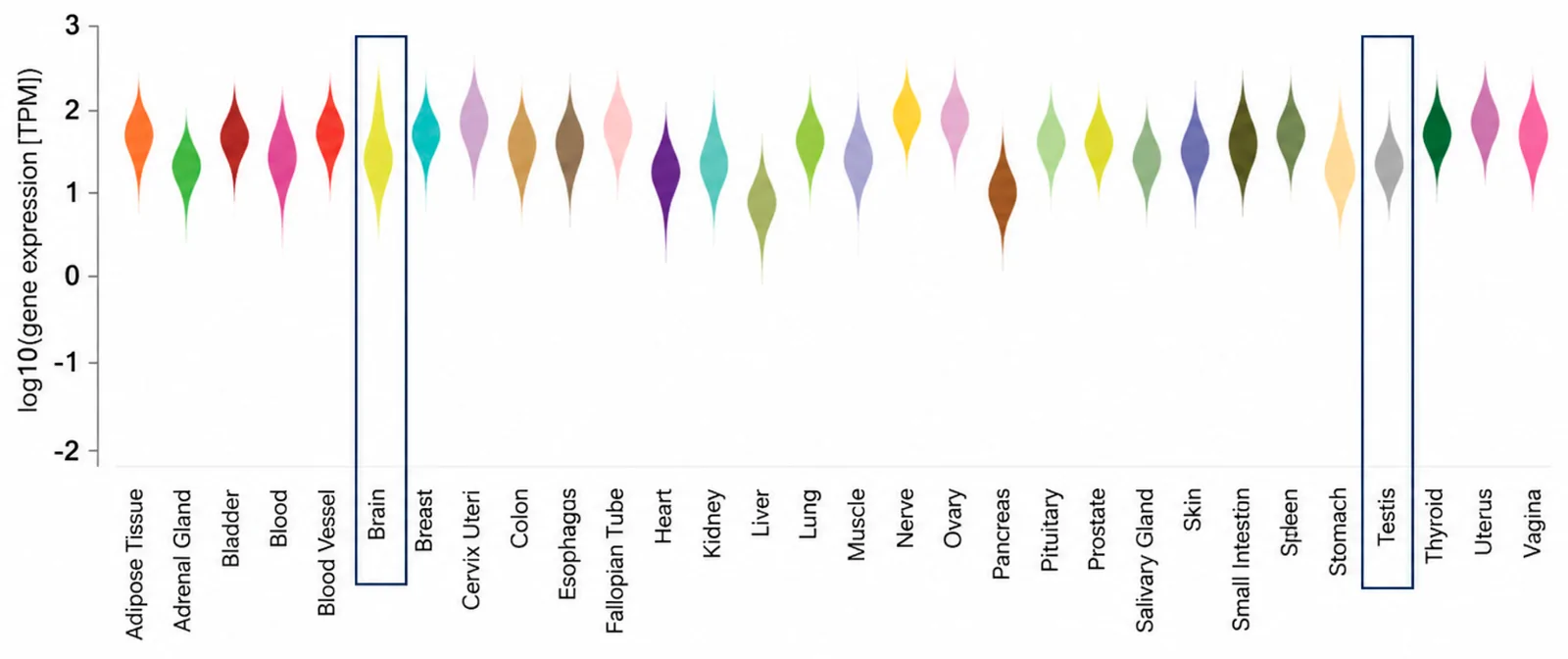
*EZH1* expression across normal human tissues. Violin plots show the distribution of *EZH1* mRNA expression (log10 transcripts per million [TPM]) across normal human tissues, as visualized using the PsychSCREEN portal [4], which integrates publicly available RNA-sequencing datasets. *EZH1* is broadly expressed across multiple tissues, with relatively high expression in the brain and testis. The elevated expression in neural tissues is consistent with the established role of EZH1 in neurodevelopment, whereas its expression in the testis suggests a role in germ-cell development and reproductive tissue homeostasis.

**Figure 7:**
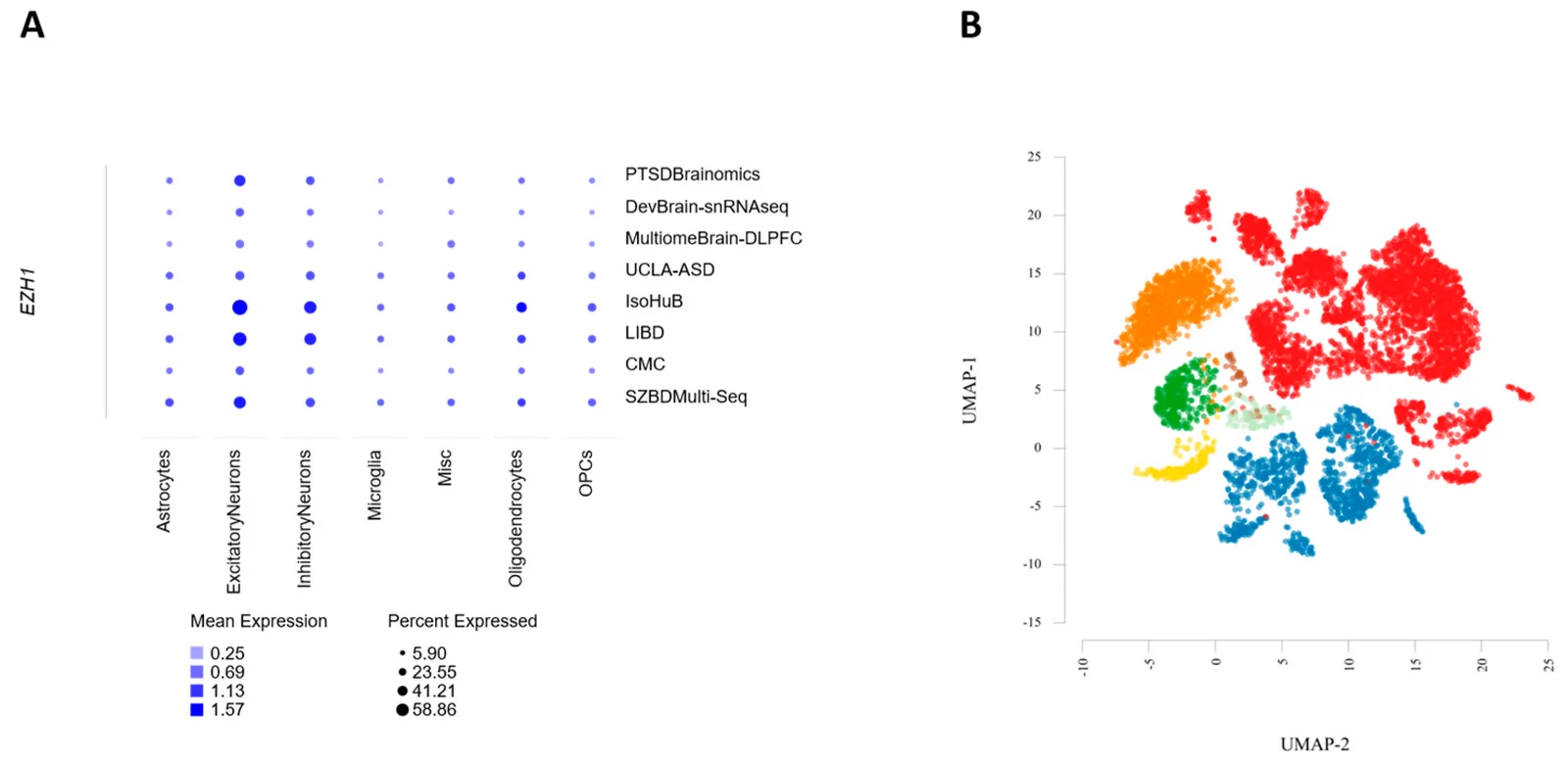
Consistent cell-type-specific expression of *EZH1* across independent human brain single-cell datasets. (**A**) A dot plot summarizing *EZH1* expression across major brain cell populations using single-cell/single-nucleus RNA sequencing datasets integrated in the PsychSCREEN database (PTSDBrainomics, DevBrain-snRNAseq, MultiomeBrain-DLPFC, UCLA-ASD, IsoHuB, LIBD, CMC, and SZBDMulti-Seq). Dot color indicates the average *EZH1* expression level, whereas dot size represents the proportion of cells expressing *EZH1*. Across independent datasets, *EZH1* is consistently expressed in multiple neuronal and glial cell populations; the highest expression is observed in excitatory neurons, supporting its role in neuronal function and cortical development. (**B**) UMAP visualization of the major cortical cell populations in the SZBDMulti-Seq dataset (*n* = 72), illustrating the cellular composition used for the single-cell expression analysis.

According to the PsychSCREEN database, *EZH1* is broadly expressed in the human brain, with relatively high expression in the cerebellum, cerebral cortex, and other brain regions. In addition, *EZH1* is expressed in several non-neural tissues, including the lung, liver, kidney, and gonads, with particularly high expression in the testis (Figure 6). Single-cell transcriptomic datasets integrated within PsychSCREEN further demonstrate that *EZH1* is expressed across multiple cortical cell populations, including excitatory and inhibitory neurons, astrocytes, oligodendrocytes, oligodendrocyte precursor cells, microglia, and vascular-related cells, with the highest expression observed in excitatory neurons (Figure 7). These findings are consistent across several independent datasets, supporting a broad role for EZH1 in the human cerebral cortex.

The high expression of EZH1 in neural tissues is consistent with its established role as a catalytic component of polycomb repressive complex 2 (PRC2), which regulates H3K27 trimethylation and is essential for neuronal differentiation, cortical development, synaptic maturation, and maintenance of neuronal identity. The prominent expression of *EZH1* in the testis also suggests a role in germ-cell development and reproductive tissue homeostasis through epigenetic regulation. In our patient, the reduced *EZH1* mRNA expression supports a loss-of-function mechanism, which may impair PRC2-mediated chromatin regulation and contribute to the observed neurodevelopmental phenotype. These findings are consistent with recent reports demonstrating that both gain-of-function and loss-of-function variants in EZH1 can disrupt normal neurodevelopment and cause neurodevelopmental disorders.

As summarized in Table 4 and Supplementary Table S1, there are overlapping clinical features among the EZH1-associated NDD patients, such as DD and ID. The unique features reported in this study are precocious puberty and lab results of persistent hypoalbuminemia and elevated GGT (Figure 1A,B). Although persistent hypoalbuminemia and elevated GGT were observed in one affected individual, further studies are required to determine whether these findings are directly related to EZH1 deficiency or represent secondary consequences of chronic medical comorbidities. Moreover, patient 2 also suffered from electrolyte variations and occasional mild anemia though she was negative for hepatitis surface antigen, hepatitis B antibodies, MRSA, C. difficile, and HIV. She had ICU stays and multidisciplinary care, receiving therapeutic management including anticoagulants, antihypertensives, broad-spectrum antibodies, systemic antifungals, and anticonvulsants (levetiracetam, phenytoin, diazepam). She had physiotherapy focusing on chest physiotherapy, balance training, and range-of-motion exercises.

In summary, we describe a novel homozygous loss-of-function *EZH1* variant associated with neurodevelopmental disorder and central precocious puberty. Our molecular findings, together with RT-qPCR and PsychSCREEN expression analyses, further expand the clinical, molecular, and phenotypic spectrum of EZH1-associated neurodevelopmental disorders and provide additional evidence supporting a loss-of-function disease mechanism.

## 4. Materials and Methods

### 4.1. Participants

Male and female patients from a consanguineous Saudi family with a total of eight members were recruited at King Faisal Specialist Hospital and Research Center (KFSHRC). The family consented by signing a consent form approved by the institutional review board (KFSHRC Research Advisory Council, RAC# 2120 022). Peripheral blood samples were collected from affected and unaffected members into EDTA and sodium heparin tubes.

### 4.2. Patient-Derived Cell Line Generation

For each member in the studied family, cell lines were prepared from sodium heparin samples using an Epstein–Barr virus (EBV)-transformed lymphoblast protocol. Blood from each family member was diluted with an equal amount of 1× PBS. Then, the diluted blood was layered onto Ficoll-Paque gradient media and centrifuged for 20 min at 3000 RPM. The buffy coat was collected and washed with 1× PBS. RPMI medium was prepared by mixing 6% penicillin/streptomycin, 0.2% normacin, 15% heat-inactivated fetal bovine serum, and 6% L-Glutamine (200 mM). The medium was prewarmed in a water bath at 37 °C before usage. The cell pellet, 2.5 mL heated medium, 2 µL cyclosporine, 6 µL PHA (phytohemagglutinin), and 500 µL pyothorax-associated lymphoma (PAL) of EBV (Epstein–Barr virus) were added into a 25 mL flask and fed with medium every other day until the cells reached confluency. For cell harvesting, we used centrifugation at 3000 RPM for 5 min.

### 4.3. DNA and RNA Extraction

A Gentra^®^ Puregene DNA Purification Kit (Gentra Systems, Inc. Minneapolis, MN, USA) was used to isolate genomic DNA (gDNA) from the collected EDTA tubes according to the manufacturer’s instructions. The quantity and quality of the isolated gDNA were measured using Nanodrop (ND-1000) (ThermoFisher Scientific Corp., Waltham, MA, USA). Cultured lymphocytes were processed to extract total RNA using a QIAamp^®^ RNA Blood Mini Kit (QIAGEN^®^, Hilden, Germany).

### 4.4. Whole-Exome Sequencing

Whole-exome sequencing (WES) was performed on the patients’ DNAs using the Illumina HiSeq2000 platform and TruSeq v3 chemistry (Illumina, San Diego, CA, USA). The sequenced data were analyzed by correlating a normal human genomic database with a pathogenic mutation database, along with the clinical phenotypes of the affected probands. The identified variant sites were annotated using population frequencies, conservation scores, disease databases, and splice predictions.

### 4.5. Filtration of Variant

Variants identified by whole-exome sequencing were filtered and prioritized using previously published approaches and established analysis pipelines [17,18,19,20,21,22,23,24]. Briefly, common population variants (minor allele frequency > 1% in the gnomAD and ExAC databases), low-quality variant calls, and variants predicted to have no functional consequence were excluded. The remaining variants were prioritized based on inheritance pattern, zygosity, read depth, variant quality, predicted functional impact on the encoded protein, and localization within coding exons. Candidate variants were further evaluated using public databases, including ClinVar, HGMD, and PubMed, and correlated with the patients’ clinical phenotypes. Pathogenicity was assessed according to ACMG/AMP guidelines using multiple in silico prediction tools before the final candidate variant was selected for clinical interpretation.

### 4.6. Structural Analysis of the Variant

The effects of the variant on protein structure and function were analyzed using PyMOL TM 3.1.6.1. [25], based on the PDB entry 7TD5 [26].

### 4.7. PCR and Gel Electrophoresis

PCR was used to confirm the WES results by amplifying gDNA samples using exon-specific forward and reverse primers (Figure 1B) for *EZH1*, which were designed by using the publicly available Primer3 algorithm. The DNA products were loaded and visualized on agarose gel to assess the performance of PCR.

### 4.8. RT-qPCR

Patient and control samples were evaluated for their relative expression of the *EZH1* gene using RT-qPCR. Since the patients’ ages and sexes were different, age-/sex-matching controls were selected accordingly. The experiments for each patient were set out differently and run on an Applied Biosystems StepOnePlus Real-Time PCR analyzer (Thermo Fisher Scientific Corp.). Nanodrop was used to assess the RNA concentration and purity. Complementary DNA (cDNA) was synthesized from extracted RNA (patient-derived cell lines) as previously published [27,28,29,30,31,32]. The variant site was targeted by *EZH1*-specific forward (5′- GCGTGGACTTAAGAAGCACC-3′) and reverse (5′-CGATCCCCACGTACTTGAGA-3′) primers. The analysis used the housekeeping gene *GAPDH* as an internal control to normalize the gene expression level. The comparative Ct (ΔΔCt) method was used to calculate relative expression levels of *EZH1*, as published before [30,33,34,35,36,37]. The output data from the experiment were analyzed by comparing the normalized expression values between control and patient samples.

## Figures and Tables

**Table 1:** Clinical features of the male patient at 6 years of age according to human phenotype ontology codes.

	Proband Phenotype	
Age (years)	8 years, evaluated at 6 years	
Gender	Male	
Variant	EZH1:NM_001321079.1:c.2109T>G:p.(Tyr703*)	
Zygosity	Homozygous	
Clinical features	Delayed ability to sit	HP:0025336
	Delayed ability to walk	HP:0031936
	Delayed speech and language development	HP:0000750
	Hyperactivity	HP:0000752
	Intellectual disability	HP:0001249
	Motor delay	HP:0001270
	Long penis	HP:0000040
	Precocious puberty	HP:0000826

**Table 2:** Summary of *EZH1* variants and associated clinical features across studies.

	This Study *	Jangam et al. (2023) [8]	Gracia-Diaz et al. (2023) [5]	Okamoto, N. et al., 2024 [9]
Number of patients	2	1	19	2
Gender	F, M	M	F, M	F
Consanguinity	Yes	NA	NA	No
Variant (cDNA)	c.2109T>G	c.2033G>C	Multiple: c.1217G>A, c.772C>T, c.1453G>T, c.1137C>T, c.2182A>G, c.2191C>G, c.2203C>T, c.1835A>T, c.1314A>C	c.865C>T c.1512C>A
Variant (protein)	p.Tyr703*	p.Ala678Gly	Multiple: p.Arg406His, p.Arg258Ter, p.Ala678Gly, p.Arg728Gly, p.Glu485Ter, p.Gln413Ter, p.Gln731Glu, p.Leu735Phe, p.Lys612Met, p.Glu438Asp	p.Gln289* p.Cys504*
Variant type	Biallelic loss-of-function (stop-gain)	Missense (gain-of-function)	19 patients (9 with biallelic loss-of-function variants, including nonsense/splice/deletion; 10 with heterozygous missense variants)	Biallelic loss-of-function (compound heterozygous nonsense variants)
Zygosity	Homozygous	De novo (heterozygous)	Homozygous, heterozygous	Compound heterozygous
Cognitive impairment	Delayed	Delayed	Delayed	Delayed
Intellectual disability	Yes	Yes	Yes	Yes
Language	Delayed	Delayed	Normal, delayed, absent	Delayed
Motor difficulties	Yes	Yes	Yes, no, NA	Yes
Central precocious puberty	Yes	No	No	Yes
MRI findings	Normal	NA	Normal, NA	Enlarged pituitary gland/ normal

Abbreviations: NA = not available; F = female; M = male. * Features of the male patient at 6 years of age according to human phenotype ontology codes.

**Table 3:** Pathogenicity assessment of the *EZH1* variant.

FEATURE	DESCRIPTION
GENE	EZH1
TRANSCRIPT	NM_001321079.1
GENOMIC LOCATION	Chromosome 17: 42703747 A>C
CDNA CHANGE	c.2109T>G
PROTEIN CHANGE	p.(Tyr703*)
VARIANT TYPE	Nonsense (stop-gain)
AFFECTED PROTEIN DOMAIN	SET methyltransferase domain
PROTEIN LENGTH/TRUNCATION	Premature stop at aa 703 of 747 aa
PREDICTED MOLECULAR CONSEQUENCE	Truncated protein or nonsense-mediated mRNA decay
POPULATION FREQUENCY	Not detected in gnomAD, ExAC, ESP, 1000 Genomes, CGMdb databases
SEGREGATION EVIDENCE	Homozygous in both affected individuals; parents heterozygous carriers
COMPUTATIONAL PREDICTIONS	CADD = 34 (deleterious); DANN = 0.99; BayesDel_addAF = 0.53; BayesDel_noAF = 0.52; FATHMM-MKL = 0.77
EVOLUTIONARY CONSERVATION	Highly conserved tyrosine residue across vertebrate species
GENE FUNCTION	Catalytic subunit of PRC2 complex responsible for H3K27 methylation and epigenetic transcriptional repression
DISEASE ASSOCIATION	EZH1 variants previously associated with neurodevelopmental disorders with developmental delay and intellectual disability
FUNCTIONAL INTERPRETATION	Predicted loss of catalytic methyltransferase activity due to truncation within the SET domain
ACMG EVIDENCE CODES	PVS1 (null variant in LOF gene); PM2 (absent from general population databases); PP3 (this is also supported by being absent in local databases) (multiple computational tools support deleterious effect)
ACMG CLASSIFICATION	Pathogenic

**Table 4:** A clinical feature comparison between previously reported patients and the patients characterized in this study.

CLINICAL FEATURES	PATIENT 1	PATIENT 2	REPORTED EZH1 CASES [5,8,9]
DEVELOPMENTAL DELAY	Yes	Yes	Common
INTELLECTUAL DISABILITY	Yes	Severe	Common
MOTOR DELAY	Yes	Yes	Reported
SPEECH DELAY	Yes	Yes	Reported
HYPERACTIVITY	Yes	Severe hyperactivity	Occasional
EPILEPSY	No	Yes	Occasionally reported
CEREBRAL PALSY	No	Yes	Rare
PRECOCIOUS PUBERTY	Yes	Not reported	Reported in some cases
BEHAVIORAL IMPAIRMENT	Mild	Severe	Variable
HYPOALBUMINEMIA	Not reported	Present	Not described
ELEVATED GGT	Not reported	Present	Not described

## Data Availability

The datasets for this article are not publicly available due to concerns regarding participant/patient anonymity. Requests to access the datasets should be directed to the corresponding author.

## Supplementary Materials

The following supporting information can be downloaded at https://www.mdpi.com/article/10.3390/ijms27167087/s1.

## Abbreviations

The following abbreviations are used in this manuscript:

ACMG	American College of Medical Genetics and Genomics
ADHD	Attention-deficit hyperactivity disorder
AA	Amino acid
ASD	Autism spectrum disorder
DD	Developmental delay
ESP	Exome Sequencing Project
EZH1	Enhancer of zeste homolog 1
F	Female
GGT	Gamma-glutamyl transferase
GOF	Gain of function
H3K27	H3 lys27
HIV	Human immunodeficiency virus
ICU	Intensive care unit
ID	Intellectual disability
KFSHRC	King Faisal Specialist Hospital, and Research Centre
LOF	Loss of function
M	Male
MRI	Magnetic resonance imaging
MRSA	Methicillin-resistant Staphylococcus Aureus
NA	Not available
NCBI	National Center for Biotechnology Information
NDD	Neurodevelopmental disorder
NT	Nucleotide
PCR	Polymerase chain reaction
PRC2	Polycomb repressive complex 2
SNP	Single-nucleotide polymorphism
TLA	Three-letter acronym
