# Supplementary material for: Biallelic EZH1 Nonsense Novel Variant in Two Siblings with Neurodevelopmental Disorder and Central Precocious Puberty: A Case Report from a Consanguineous Saudi Family
Source: Int J Mol Sci. 2026 Aug 7;27(16):7087. doi: 10.3390/ijms27167087 (PMC13512644; doi:10.3390/ijms27167087)
Supplement: Supplementary file 1 [file ijms-27-07087-s001.zip › ijms-4380283-supplementary.pdf]

Supplementary Table S1. Clinical and molecular comparison of the present cases with previously reported EZH1-associated neurodevelopmental disorder cases.

Clinical / Molecular Feature	*Current Study*	*Jangam et al., 2023*	*Gracia-Diaz et al., 2023*
**Number of affected individuals**	2 siblings	1 individual	19 individuals
**Inheritance / zygosity**	Homozygous; autosomal recessive	De novo heterozygous	Biallelic truncating and heterozygous missense
**Variant type**	Nonsense / stop-gain	Missense	Nonsense, splice/deletion, and missense
**Protein change**	p.Tyr703*	p.Ala678Gly	Multiple, including p.Arg258Ter, p.Glu485Ter, p.Gln413Ter, p.Ala678Gly, p.Arg728Gly, p.Gln731Glu, p.Leu735Phe
**Predicted / demonstrated mechanism**	Loss-of-function; reduced EZH1 transcript expression	Gain-of-function; increased H3K27me3 in Drosophila	Recessive LOF and dominant GOF mechanisms
**Developmental delay**	Present	Present	Present
**Intellectual disability / cognitive impairment**	Present	Present / developmental impairment	Present; variable severity
**Speech / language delay**	Present	Present	Present; variable
**Motor delay / motor impairment**	Present	Present	Present; variable
**Hypotonia / muscle weakness**	Not clearly reported	Hypotonia and proximal muscle weakness	Variable / not universal
**Epilepsy / seizures**	Present in Patient 2; absent in Patient 1	No seizures reported	Variable
**MRI findings**	Normal in Patient 1; not clearly available for Patient 2	Mild prominence of ventricles / extra-axial spaces; otherwise unremarkable	Mild or unremarkable in most evaluated individuals
**Behavioral abnormalities**	Hyperactivity / behavioral impairment	Dyskinesia and neurodevelopmental abnormalities	Autism, aggressive behavior, or obsessive-compulsive features in some individuals
**Growth**	No overgrowth; Patient 1 with precocious puberty	Short stature / low-normal height	No consistent overgrowth; variable stature
**Central precocious puberty**	Present in Patient 1	Not reported	Reported in some biallelic EZH1 cases
**Additional biochemical findings**	Hypoalbuminemia and elevated GGT in Patient 2	Not reported	Not reported
**Main contribution**	Novel homozygous EZH1 p.Tyr703* variant with segregation, structural, and RT-qPCR support	Functional validation of EZH1 p.A678G GOF in Drosophila	Established EZH1-associated dominant and recessive NDD spectrum

Abbreviations. GGT: gamma-glutamyl transferase; GOF: Gain-of-function; H3K27me3: Trimethylation of histone H3 lysine 27; LOF: Loss-of-function; MRI: Magnetic resonance imaging; NDD: Neurodevelopmental disorder; RT-qPCR: Reverse transcription quantitative polymerase chain reaction.
